# *Wohlfahrtia nuba* (Wiedemann, 1830) (Diptera: Sarcophagidae) Development and Survival Under Fluctuating Temperatures

**DOI:** 10.3390/insects16060628

**Published:** 2025-06-13

**Authors:** Abeer S. Yamany, Manal F. Elkhadragy, Rewaida Abdel-Gaber

**Affiliations:** 1 Department of Zoology, Faculty of Science, Zagazig University, Zagazig 44519, Egypt; 2 Department of Biology, College of Science, Princess Nourah bint Abdulrahman University, Riyadh 11564, Saudi Arabia; 3 Department of Zoology, College of Science, King Saud University, Riyadh 11451, Saudi Arabia

**Keywords:** forensic entomology, postmortem interval, development rate/time, temperature, Immature stages, *Wohlfahrtia nuba*

## Abstract

The flesh fly, *Wohlfahrtia nuba* (Wiedemann) (Diptera: Sarcophagidae), is one of the first necrophagous insects to arrive on a cadaver and is vital for understanding decomposition. Environmental factors, especially temperature, influence insect development, which is crucial for estimating postmortem interval (PMI) in forensic entomology. This study explored how seasonal temperature variations affect the survival and development of *W. nuba*’s immature stages. The *W. nuba* colony was reared in the laboratory for four seasons from 3 October 2023 to 30 September 2024. The duration of the larval and pupal phases, the percentage of survival and mortality of the larvae and pupae, the larval growth rate, the percentage of emergence, fecundity, the sex ratio, and the pre-larviposition period were among the many life cycle characteristics that were documented during the study. Research indicates that seasonal changes affect development, shortening the growth period as temperatures rise. Flies raised at an average temperature of 38.3 °C grew faster but experienced higher larval mortality and lower survival rates. The average duration of larval and pupal stages was reduced, with an optimal development temperature of 27.9 °C showing higher survival rates, maximum body weight, and fecundity. The largest mortality rate occurred during winter at an average temperature of 18.5 °C, with males and females showing significant pupal elongation. The findings could help forensic entomologists working on legal investigations to ascertain PMI.

## Simple Summary

This study investigates the impact of seasonal temperature variations on the survival and development of *Wohlfahrtia nuba*. *Wohlfahrtia nuba*, a species of flesh fly, is a critical indicator for the rapid detection of deceased bodies and is one of the first necrophagous insects to inhabit cadavers. *Wohlfahrtia nuba* is widely distributed across various countries in Asia and Africa, including Saudi Arabia, Pakistan, Iran, and the nations along the western shore of the Red Sea, as well as Egypt, Guinea, Libya, Mauritania, Senegal, and Tunisia. Temperature influences the development of *W. nuba* and is essential for estimating the postmortem interval in forensic entomology. Our research indicated that seasonal variations reduce the growth duration as temperatures increase, with flies raised at an average temperature of 38.3 °C exhibiting accelerated growth but facing elevated larval mortality and diminished survival rates. The optimal development temperature of 27.9 °C increased survival rates, maximum body weight, and enhanced fecundity. The highest mortality rate was observed in winter at an average temperature of 18.5 °C, with males and females exhibiting notable pupal elongation. The findings may aid forensic entomologists in legal investigations to determine the postmortem interval.

## 1. Introduction

Flesh flies, belonging to the family Sarcophagidae, have received considerable interest in recent years for their role as critical indicators of human decomposition. These flies can detect a deceased body within minutes following death, even from several kilometers away [1,2,3]. Flesh flies also play an essential role in the food chain and have been recognized for their medical and forensic significance [4]. *Wohlfahrtia nuba* is a flesh fly distributed extensively in Africa and Asia [5,6]. The species *W. nuba*, with potential forensic value, can aid in estimating PMI [7]. Numerous studies, including those conducted in urban environments, have found that adult flies from the Sarcophagidae and Calliphoridae families can locate decomposing bodies, highlighting their role in forensic investigations [7,8,9,10,11,12,13,14,15,16,17,18].

Forensic entomology plays a crucial role in criminal investigations by accurately determining the developmental duration of immature flesh flies found on a deceased body. This information is essential for calculating the minimum postmortem interval (mPMI). Flesh flies undergo variations in their growth and development due to various environmental temperatures, food availability, relative humidity, photoperiod, and carcass decomposition. However, temperature is the most crucial factor influencing the development of forensically important species [19,20,21,22]. Therefore, it is better to utilize data collected at a temperature close to the place of death [23,24]. Temperature fluctuations can accelerate or slow down developmental stages, depending on the species and specific stage of development [22,23,24,25,26,27,28,29,30,31,32]. Therefore, temperature significantly influences the estimation of mPMI and should be considered when gathering larva samples from decomposed bodies to accurately determine the time of death [33]. Previous studies have extensively documented the development of *W. nuba* [34,35], as well as *W. magnifica* [36] and other species within the Sarcophagidae and Calliphoridae families [37,38,39,40,41] in various biogeographic regions.

Analyzing the effects of seasonal temperature variations is crucial for forecasting the abundance and distribution of insect species, as temperature markedly affects essential demographic processes, including development and mortality, since they are ectothermic organisms. Accurate estimates of the duration of immature stages are necessary for estimating mPMI; however, data on the developmental duration of *W. nuba* are limited. Accordingly, this study aims to investigate how temperature fluctuations affect the duration of developmental stages in *W. nuba*, a flesh fly species that holds great importance in forensic investigations. The findings of this research are anticipated to be informative and valuable in understanding the developmental process, providing a comprehensive understanding of its biology and developmental duration. This will establish baseline biological data that can assist forensic experts in more accurately estimating mPMI under conditions specific to the Kingdom of Saudi Arabia.

## 2. Materials and Methods

### 2.1. Sampling Location and Experimental Design

Adult flies were obtained from a colony stock kept for ten generations under laboratory conditions at the University College Biology Laboratory, Hafr Al Batin University, Saudi Arabia. The stock colony of *W. nuba* was established from larvae collected from rabbit carcasses weighing 1300 to 1500 g in March 2023 in Qaryat Al Ulya Province, Eastern Region, Saudi Arabia, for a previous study, with identification based on morphological characteristics of the larvae and COI gene molecular sequencing. To maintain the heterogeneity of the colony and avoid inbreeding, wild-caught flies were periodically introduced into the established laboratory colony.

### 2.2. Laboratory Rearing of Wohlfahrtia nuba Colony

**Figure 1:**
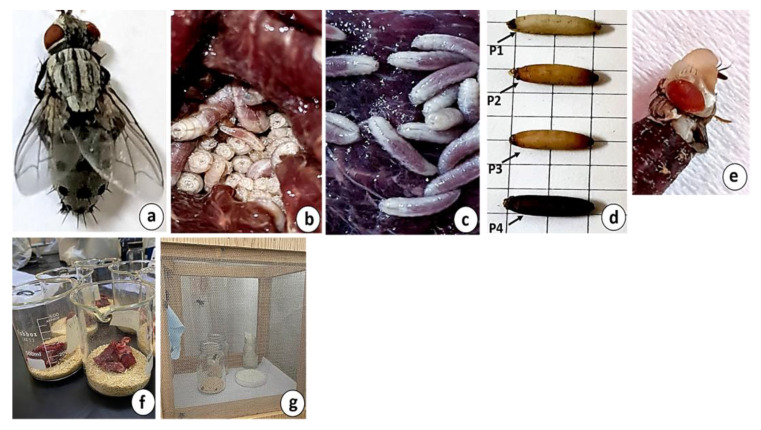
Illustration of *Wohlfahrtia nuba*. (**a**) Dorsal view of the adult female; (**b**,**c**) second and third larval instars; (**d**) pupal stages of P1–P4, where P1 exhibits a white puparium, P2 displays a light brown puparium, a brown puparium characterizes P3, and P4 features a dark brown puparium; (**e**) adult eclosion; (**f**) larval rearing beakers; (**g**) adult rearing cage.

Adult flies were placed in 30 × 30 × 30 cm rearing cages (Figure 1g). Adult flies were provided with granulated sugar, water, and powdered milk, and allowed to mate and lay larvae. The water was supplied via a cotton wick inserted into a water bottle. A plastic cup with approximately 60 g of fresh beef was positioned in the rearing cage as a substrate for larval deposition. It was replaced daily with another cup containing 60 g of fresh meat. The cup was observed every 12 h to monitor the deposition of larvae over two weeks. The larvae were transferred to a 500 mL glass beaker with a 2 cm layer of sawdust at the bottom (Figure 1f). The beaker was covered with muslin and secured using a rubber band. The first instar larvae were given a small piece (30 g) of minced fresh bovine liver to prevent decay of uneaten portions and avoid the larvae becoming stuck to spoiled liver. As the larvae grew, the quantity was gradually increased to 45 g for the second instar larvae and 60 g for the third instar larvae. Larvae were fed each morning until they reached the pupal phase. Newly formed pupae were carefully collected and placed in a glass bottle, which was then transferred to a rearing cage for adult emergence (Figure 1g).

The present study was conducted from 3 October 2023 to 30 September 2024, during which the fly colony was housed indoors in a well-ventilated insect chamber. This setup allowed for the monitoring of fly colonies across all four seasons. This chamber, located behind the laboratory on the ground floor, features several open windows to enhance natural ventilation. A digital thermometer and hygrometer were used to continuously record the internal temperature and relative humidity, with measurements recorded every 12 h throughout the test. Furthermore, the internal temperature and humidity measurements were compared with data from an external weather station to ensure that the conditions experienced by the fly colonies accurately reflected seasonal variations. Summer temperatures averaged 38.3 °C (range: 30.66–46.83 °C), relative humidity averaged 17.23%, and the photoperiod was 14 h of daylight and 10 h of darkness. Autumn temperatures averaged 35.8 °C (range: 27.35–43.5 °C), relative humidity averaged 21.36%, and the photoperiod was 12 h of daylight and 12 h of darkness. Winter temperatures averaged 18.5 °C (range: 13.65–22.83 °C). Relative humidity averaged 41.39%, and the photoperiod was 10 h of daylight and 14 h of darkness. Spring temperatures averaged 27.9 °C (range: 17.11–29.47 °C), the relative humidity averaged 26.97%, and the photoperiod was 12 h of daylight and 12 h of darkness.

### 2.3. Developmental Duration

#### 2.3.1. Larval Stage

In the rearing cage, the adult flies were provided with a plastic cup containing approximately 60 g of fresh beef and were observed every hour for larval laying. Using a fine brush, 500 recently laid larvae were collected immediately after they were laid and then divided into five groups to determine the duration of the larval stage. Each group contained 100 larvae to prevent competition between the larvae that could hinder their growth and to account for any possible growth stimulation caused by the heat generated by the larvae. These groups were placed in separate 500 mL glass beakers, each with a 2 cm layer of sawdust at the bottom (Figure 1f). The larvae in each group were reared and fed as previously described. The larvae were checked every 12 h as they moved through the sawdust until they reached the pupal stage. A meticulous monitoring process was used to record daily larval mortality, including visual assessments to minimize disturbance to their development. In cases where deceased larvae were identified, they were gently extracted to prevent any potential impact on the remaining population. The experiment was repeated three times per season.

#### 2.3.2. Pupal Stage

At the end of their feeding phase, the larvae entered the prepupae phase and moved away from their food source. Each day, newly formed pupae were carefully collected from each group and placed in separate sample tubes to await adult emergence. The pupae were counted. Sample tubes were monitored every 12 h until the adults emerged, allowing for the determination of the pupal stage duration and the calculation of pupal survival and mortality rates. For each season, the experiment was repeated three times.

#### 2.3.3. Adult Stage

Female fecundity and pre-larviposition periods were determined using five pairs of adults. Each pair consisted of a virgin female and a one-day-old male kept in a 15 × 15 × 15 cm cage, according to the study by Li et al. [42]. Sucrose and fresh meat were provided. Cages were checked twice a day for larva laying. The experiment was repeated three times per season.

### 2.4. Measurements

Five larvae from each larval stage and five prepupae were carefully collected in the morning from each previously described group that was designed for the developmental duration experiment and washed with water at laboratory temperature. The larvae and prepupae were then euthanized by submerging in near-boiling water for 30 s. The lengths and weights of specimens were recorded after being submerged in 80% ethanol. As per the study conducted by Adams and Hall [43], it was observed that there was a negligible change of approximately 1.2% in length during the first hour of preservation in 80% ethanol. Once the larvae transitioned to the pupal stage, the lengths and weights of five pupal specimens from each group were measured.

Weights were measured using an electronic balance with high accuracy (GR-200 balance; A&D Company, Limited, Tokyo, Japan; accuracy, 0.1 mg). The length measurements were taken using an ocular micrometer attached to a light microscope (Olympus U-CMAD3 BX50, Tokyo, Japan).

### 2.5. Statistical Analysis

Results are expressed as mean ± S.E. One-way analysis of variance (ANOVA) and the LSD test were used to analyze seasonal fluctuations in immature development rates and to determine the difference in means. The influence of temperature fluctuations on immature stage development rates was examined using linear regression. Statistical analyses were performed with IBM’s SPSS software, particularly Windows Assessment version 26.0.

## 3. Results

### 3.1. The Effects of Temperature Fluctuations Throughout the Four Seasons on the Larval Growth Rate and Duration of Immature Stages of W. nuba

#### 3.1.1. Larval and Pupal Durations and Total Developmental Duration

As shown in Table 1, temperature significantly affected larval and pupal duration. A significant negative correlation was observed between temperature and larval duration (R = 0.91, R^2^ = 0.83). Based on the findings of the ANOVA test (F-value = 859.82; *p* = 0.00), there is a significant decrease in the length of larval stages as the temperature rises. The larvae raised at an average temperature of 18.5 °C exhibited the longest duration (10.28 ± 0.07 days), while the shortest duration was observed in larvae reared at an average temperature of 38.3 °C (3.81 ± 0.04 days). This relationship was determined using a linear regression model in which the larval duration was influenced by the temperature (12.09–2.25 × temperature). The larvae pupated on day 4 in summer at an average temperature of 38.3 °C and in autumn at an average temperature of 35.8 °C. However, pupation was not observed until day 7 in the spring at an average temperature of 27.9 °C. In winter, at an average temperature of 18.5 °C, pupation was delayed until day 10. The average duration of larval stages was shorter than the average duration of pupal stages (*p* < 0.05). The duration of pupation was reduced by half as the temperature increased from spring to summer.

Moderate negative correlations were observed between temperature and durations of female and male pupae, with correlation coefficients of 0.54 and 0.61. There was a significant decrease in pupation duration for both female and male specimens over time (ANOVA test; F value = 164.75 and 131.62, respectively; *p* = 0.00). During summer, there was no significant difference in pupal duration between females (8.69 ± 0.12 days) and males (8.88 ± 0.19 days). Significant elongation was observed during the winter for both female (62.57 ± 7.04) and male (64.83 ± 5.80) pupae. Moreover, there were no significant differences in female pupation duration between spring and autumn (*p* = 0.09) or between autumn and summer (*p* = 0.72). Pupae reared at an average temperature of 38.3 °C had the shortest duration (linear regression model: female pupal duration = 43.64–10.61 × temperature, male pupal duration = 65.15–17.04 × temperature).

Table 1 shows the overall developmental duration from larvae laid to emergence at each of the four seasonal temperatures, with the shortest being in autumn (12.33 and 12.68 days for female and male immature, respectively) and the longest being in winter (72.17 and 73.50 days for female and male immature, respectively). Under extreme temperatures, there was a noticeable increase in the development speed, resulting in shorter durations of immature stages, including larval and female or male pupal stages. There was a moderate negative correlation between temperature and developmental duration (R = 0.64 for female immatures and 0.59 for male immatures). The results of the statistical analysis (ANOVA test; F-value = 152.51, 106.73 for female and male immature, respectively; *p* < 0.05) confirm a significant and gradual decrease in developmental duration with increasing temperature (linear regression model: total developmental duration for female immature = 62.19–14.43 × temperature; total developmental duration for male immature = 55.89–12.80 × temperature). The difference in duration between female and male immatures was not significant in spring (*p* = 0.59), winter (*p* = 0.89), and summer (*p* = 0.15), while in autumn, this difference was significant (*p* < 0.05).

#### 3.1.2. Larval Growth Rate (LGR)

**Figure 2:**
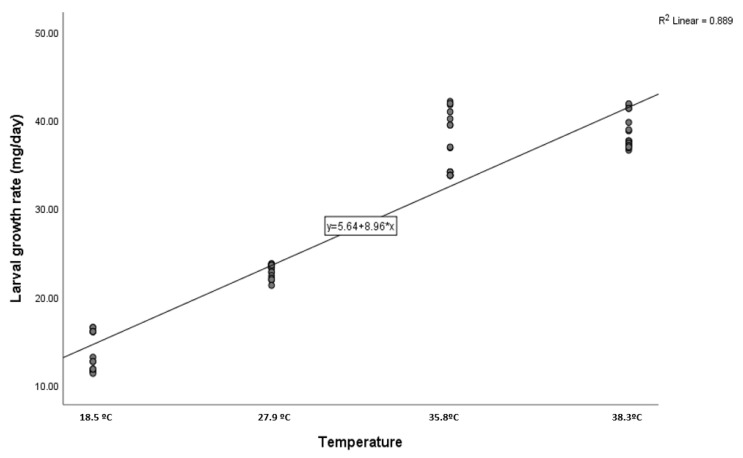
Effect of seasonal temperature variation on the growth rate of *Wohlfahrtia nuba* larvae.

The larvae gradually increased in size until they reached their maximum size (Table 2). Then, as they prepared to pupate, their length and weight decreased. Seasonal temperature variation had a significant effect on LGR. Linear regression can be used to express the equations that describe the relationship between larval growth rate (Y) and temperature (X) (larval growth rate = 5.64 + 8.96 × temperature). The data show a strong and significant correlation between temperature and LGR (R = 0.94; ANOVA test, F-value = 478.87; *p* = 0.00; Figure 2). During the summer, larval growth peaked at 38.61 ± 0.46 mg/day at an average temperature of 38.3 °C. On the other hand, larval growth rate reached its lowest value (13.46 ± 0.52 mg/day) during the winter season, coinciding with the lowest average temperature of 18.5 °C.

### 3.2. Effects of Temperature Fluctuations Throughout the Four Seasons on the Survival and Mortality of W. nuba Larvae and Pupae

Table 3 illustrates that temperature fluctuations over the four seasons significantly affect larval and pupal survival rates. Temperature inversely affects larval survival (R = 0.43; ANOVA test, F-value = 4.22; *p* = 0.01). Larval survival peaked at 95.75 ± 0.82% during spring at an average temperature of 27.9 °C. In the summer, larval survival declined as temperature rose, reaching 58.57 ± 8.96% (*p* < 0.05). Conversely, in the winter season, at an average temperature of 18.5 °C, larval survival rose significantly as temperature decreased, reaching 91.73 ± 2.02%. Larval survival rates decrease as spring, fall, and summer temperatures increase, as indicated by the linear regression model: percentage of larvae surviving = 100.34–8.98 × temperature. On the other hand, a significant correlation was observed between temperature and winter–spring larval survival (R = 0.37; ANOVA test, F-value = 4.89; *p* = 0.01).

The percentage of pupae that successfully developed into adults peaked at an average temperature of 27.9 °C, with a recorded percentage of 97.18 ± 0.44. However, with moderate correlation (R = 0.57, ANOVA test; F-value of 923.48, *p*-value = 0.00), pupal survival decreased significantly as temperature decreased across all temperature conditions tested, except for the winter season. As temperatures declined, pupal survival dropped to the lowest level of 45.73 ± 0.99. Temperature variation accounts for 70% of the variation in survivorship, as shown by the linear regression model (pupal survivorship = 53.5 + 10.12 × temperature).

Table 3 shows the mortality rates of immature stages across various temperature conditions. At an average temperature of 38.3 °C, 41.43% of the first larvae failed to reach the pupal stage. Similarly, at an average temperature of 18.5 °C, 45.67% of the pupae failed to develop into adults. The minimum larval and pupal mortality rates were 4.25% and 3.18%, respectively, observed during spring at an average temperature of 27.9 °C.

### 3.3. Effects of Temperature Fluctuations Throughout the Four Seasons on the Weight and Length of W. nuba Larvae During Each of Their Three Feeding, Prepupae, and Pupae Stages

**Figure 3:**
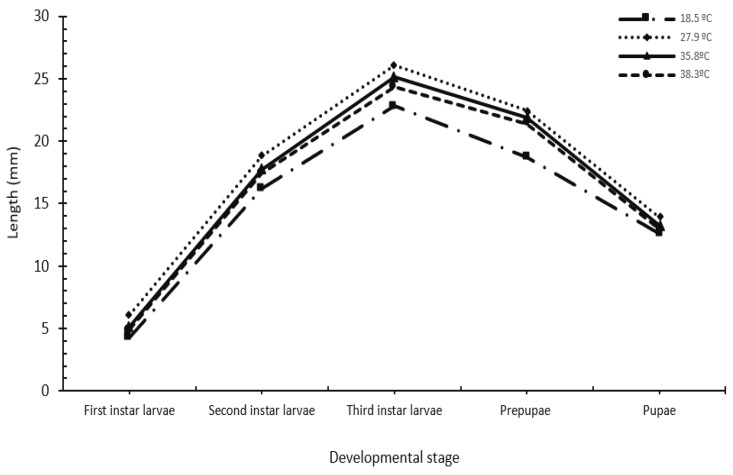
The effect of seasonal temperature variation on the length of *Wohlfahrtia nuba* larvae during the three instar larvae, prepupae, and pupae stages.

The larvae gradually increased in size and weight over time until they reached their maximum size and weight. After that, their length and weight decreased as the larvae neared pupation. There was a marked temperature-dependent variation in the weight and length of *W. nuba* larvae. As the average temperature increased from 27.9–35.8 °C to 38.3 °C, the weight of the three larval instars (feeding and wandering) gradually decreased (Table 4). In the spring season, at an average temperature of 27.9 °C, the highest weights and lengths were observed for three feeding larval instars and prepupae. The recorded weights were 17.49 ± 0.36 mg, 111.25 ± 3.92 mg, 191.82 ± 1.54 mg, and 124.95 ± 2.56 mg. The measured lengths were 6.07 ± 0.31 mm, 18.82 ± 0.49 mm, 26.11 ± 0.33 mm, and 22.44 ± 0.32 mm, respectively (Figure 3). In contrast, the three feeding larval instars and prepupae exposed to lower winter temperatures averaging 18.5 °C weighed the least (12.41 ± 0.41 mg, 70.47 ± 4.06 mg, 141.41 ± 4.02 mg, and 104.57 ± 3.36 mg, respectively) and were the shortest (4.29 ± 0.39 mm, 16.18 ± 0.37 mm, 22.82 ± 0.38 mm and 18.72 ± 0.39 mm, respectively). The weight and length of the third feeding larval instars decreased significantly under all the temperature conditions tested when they stopped feeding and entered the prepupae in search of the pupation site. 

Temperature fluctuations had a negative impact on pupal length and weight, with significant correlations observed (length: R = 0.32, F = 9.81, *p* = 0.00; weight: R = 0.56, F = 36.79, *p* = 0.00). The highest values were recorded during spring (13.91 ± 0.29 mm for pupal length and 109.71 ± 2.57 mg for pupal weight). The linear regression model was pupal length = 14.11–0.38 × temp, pupal weight = 118–0.01 × temp.

### 3.4. The Effects of Temperature Fluctuations Throughout the Four Seasons on the Percentage of Adult Emergence of Females and Males, Sex Ratio, Pre-Larviposition Period, and Fecundity of W. nuba

The data presented in Table 3 show a significant seasonal variation in the percentage of pupae that reached the adult stage. The highest percentage of pupae reached the adult stage (97.18%) was observed during the spring season at an average temperature of 27.9 °C (*p* < 0.05) and gradually decreased as temperature increased, reaching 81.64% at a high summer temperature averaging 38.3 °C (*p* < 0.05). The sex ratio of the emerged flies was determined by calculating the percentage of females to males. The ratios recorded in various seasons were 1.50:1, 1.29:1, 1.26:1, and 1.60:1 for spring, summer, autumn, and winter (Table 5). The ratios did not differ significantly across summer, autumn, spring, and winter (*p* > 0.05). The data presented in Table 5 shows that the number of larvae reaching adults and the percentage of female emergence were highest during the spring season at an average temperature of 27.9 °C, with values of 56% and 60%, respectively. As the temperature increased, these values gradually decreased, reaching 26.83% and 56.25%, respectively, at a high summer temperature averaging 38.3 °C (*p* < 0.05). 

An inverse relationship was noted between temperature and pre-larviposition period, with a strong negative correlation (R = 0.74, R^2^ = 0.54, F = 43.55, *p* = 0.00). The linear regression model was as follows: pre-larviposition period = 13.02–2.29 × temperature. More than half (54.4%) of the variation in the pre-larviposition period is attributed to temperature. In summer, the elevated temperature, averaging 38.3 °C, affects the pre-larviposition period, which is reduced to 4.17 ± 0.31 days. The duration of this period shows a gradual increase during autumn (5.76 ± 0.41) and spring (7.83 ± 0.35). The highest significant increase (11.25 ± 0.33 days) was observed in winter at an average temperature of 18 °C (Table 5).

Table 5 illustrates a slight negative correlation between female fecundity and temperature (R = 0.05, R^2^ = 0.00, F = 73.26, *p* < 0.05). The linear regression model derived from the data can be represented by the equation: fecundity = 44.54–0.23 × temperature. When examining fecundity across different seasons, the results highlight notable variations. In the spring season, the average female fecundity was recorded at a significantly elevated mean of 51.42 ± 1.22 larvae per female, indicating optimal conditions for reproduction during this time. In contrast, during the summer, there was a significant reduction (42.08 ± 0.65 larvae/female, *p* < 0.05). During the winter, female fecundity significantly declined (*p* < 0.05), with a slightly lower value of 39.58 ± 0.34.

## 4. Discussion

The results of this study show that the total developmental duration of the forensically important flesh fly, *Wohlfahrtia nuba*, fluctuates seasonally in response to temperature variations, showing a linear relationship between developmental duration and temperature, consistent with the results of [22,29,44,45,46,47,48,49,50,51]. As temperatures rise, *W. nuba* flies exhibit enhanced growth rates with higher metabolic activity, leading to more rapid development. The developmental duration is inversely correlated with the environmental temperature. These findings align with previous studies conducted by various scholars [11,29,32,44,50,51,52,53,54,55,56].

The average pupal duration was significantly longer (*p* < 0.05) than larval duration. Pupae reared at the highest temperature, averaging 38.3 °C, had the shortest developmental duration, whereas lower temperatures in winter resulted in significant lengthening of developmental duration for both male and female pupae. The lengthened duration of the pupal stage during the winter suggests that the physiological processes taking place during this time are particularly influenced by temperature. The time required for larvae and pupae to develop and emerge was consistently around 72.17 days for females and 73.50 days for males when reared at an average temperature of 18.5 °C. The shortest developmental duration was observed during autumn at an average temperature of 35.8 °C. Females required 12.33 days to develop, while males required 12.68 days. The results align with earlier research on different flesh fly species [29,50,51,57,58,59].

The developmental duration of *W. nuba* from the first instar larvae to adult emergence is influenced by rearing temperature. Previous studies reported similar results [34,35], indicating that the developmental duration ranges from 34.7 days at 21 °C to 13.6 days at 37 °C. In 2015, Kotzé et al. [50] found that larval and pupal stages were accelerated by 10.4 days at 35 °C. On the other hand, lower temperatures can prolong the duration of larval development, with a recorded duration of 53.9 days at 19 °C and 13.5 days at 27 °C, consistent with the present findings.

The present findings were consistent with a study conducted in Northern Thailand in 2003–2004, which observed the development of flies under natural temperature conditions. The results showed a seasonal variation in the developmental duration of the flies. In summer, larval development took approximately 72 h; in the rainy season, it took 72–96 h; and in winter, 96 h [36,38]. The life cycle of *Sarcophaga (Liosarcophaga*) *dux* (Thomson) in Malaysia lasted 312.0 ± 3.0 h from the first larval stage to the adult stage under uncontrolled indoor temperature conditions [60]. Sert et al. [22] investigated the developmental periods of *Sarcophaga* (*Liopygia*) *argyrostoma* (Robineau-Desvoidy). The findings indicated that the recorded development period was around 319 h (±1.41) under fluctuating temperature conditions at 25 °C. A recent study by Shang et al. [51] discovered that the developmental duration of *Sarcophaga* (*Boettcherisca*) *peregrina* (Robineau-Desvoidy) showed significant variations in response to temperature fluctuations.

There is a difference in the developmental duration from larvae laid to pupation in *W. nuba* compared with other flesh flies like *S. dux* [61] and *Sarcophaga (Liopygia*) *ruficornis* (Fabricius) [62]. Previous research found that *S. dux* took 13.6 days to develop when reared at 32 °C. Research by [63] indicates that adult *Sarcophaga* (*Robineauella*) *caerulescens* (Zetterstedt) larvae typically emerge within 8–12 days. Conversely, the authors of [29] found that elevated temperatures slowed *Calliphora vicina* (Robineau-Desvoidy) and *Calliphora vomitoria* (Linnaeus) and accelerated *S. argyrostoma* and *Lucilia cuprina* (Wiedemann). In addition, *S. argyrostoma* larvae were observed to pupate on day 15 at 13 °C. Low temperatures caused an increase in the length of *C. vicina* for 8 days, and *S. argyrostoma* larvae reared at 13 °C first pupated on day 17, whereas at high temperatures (29 °C), development to pupation was completed within 9 days. *Chrysomya albiceps* (Wiedemann) can take 8–12 days to develop at 28 °C, 25–51 days at 11 °C, and 19–27 days at 15 °C [64,65]. The authors of [45,66,67] found that *S. dux* raised at 32 °C had a developmental period of 13.6 days. In this case, the observed variation may be due to the unique climatic conditions in the Eastern Province of Saudi Arabia, which is characterized by a dry and hot atmosphere during the summer months.

Seasonal temperature variations were found to affect larval and pupal survival rates. The highest recorded larval and pupal survival rates were 95.75% and 97.18%, respectively, at an average temperature of 27.9 °C; mean larval length and weight also reached their maximums at this temperature. In winter, only 45.73% of the pupae developed into adults; however, twice as many pupae completed development at an average temperature of 35.8 °C. This observation is consistent with previous studies by [34,35] on *W. nuba*. In addition, it exceeds the results of [68], which reported an adult emergence rate of 84.44% for *Ch. albiceps* at 30 °C. The study found that the rate of survival of *Lucilia cuprina* pupae remained above 75% when exposed to temperatures below 30 °C; however, at 18 °C, a significant number of larvae died during the prepupae [69]. The authors of [29] found that only eight *S. argyrostoma* larvae hatched when exposed to a low temperature of 13 °C, and none of them could complete their development to the pupal stage. A study published by [50] in 2015 found that higher temperatures negatively influenced growth and survival rates. The larval mortality rate peaked at 41.43% at an average temperature of 38.3 °C during the summer season.

In contrast, the percentages of larvae and pupae that failed to complete development were much lower, at 4.25% and 3.18%, in the spring season at an average temperature of 27.9 °C. The average temperature range of 27.9 to 35.8 °C was optimal for rearing all stages, resulting in faster development, lower mortality, and increased weight. It is important to note that although a highly precise method was employed for monitoring and recording daily larval mortality, some disturbance to the larvae may have occurred, potentially contributing to some mortality, especially since the larval stage is more susceptible to environmental stress than the pupal stage. The larval stage is more susceptible to low temperatures than the pupal stage. Specifically, a significant number of immature stages did not survive when exposed to an average temperature of 38.3 °C for larvae and an average temperature of 18.5 °C for pupae. The data presented in this study are consistent with previous findings on *W. nuba* [35] and other medically important species, including *S. ruficornis* [62] and *Sarcophaga* (*Bercaea*) *Africa* (Wiedemann) [70]. In 2004, ref. [61] showed that *S. dux* had the highest mortality rates at 16 and 36 °C, peaking at 73% and 64%, respectively. On the other hand, the lowest mortality rates were observed at 20 °C and 24 °C, with 21% and 15%, respectively.

The highest larval and pupal weights and lengths were obtained when reared at an average temperature of 27.9 °C, with lower values observed at higher temperatures. The metabolic rate is significantly reduced at low temperatures, resulting in higher body weights. During the post-feeding stage of the larvae before pupation, there was a decrease in both body length and weight, measured as 124.95 ± 2.56 mm and 22.44 ± 0.32 mg, respectively. These results are consistent with previous studies conducted on different species at different temperatures [35,44,62,70]. When the pupal weights of *S. dux* were examined, it was found that the highest weights were observed at temperatures of 20 and 24 °C, while the lowest weights were recorded at temperatures of 16 and 36 °C [61]. Studies conducted by [51,59] found that low temperature affects the morphological characteristics of *Chrysomya*, a blow fly of forensic importance. These characteristics include length, width, and weight. 

The pre-larviposition period is inversely correlated with temperature, being highest in winter (11.25 days) and lowest in summer (3.86 days). In a study conducted by [71], the preoviposition period of *Ch. albiceps* lasted approximately 4.25 days under high-temperature conditions. The preoviposition period occurs five days after mating, with an average lifespan of 3–4 weeks, as supported by various studies [35,64,72].

Temperature had a significant effect on the fecundity of female insects, with a clear inverse relationship between fecundity and seasonal temperature variation. Fecundity peaked at an average temperature of 27.9 °C, with an average of 52.33 larvae per female. This indicates that the most suitable temperature for the highest fecundity value is 27.9 °C. These findings contradict what [73,74] found in *L. cuprina*. Similarly, [65] observed that *Ch. albiceps* had varying levels of female fecundity, depending on temperature. The highest significant fecundity rates (287.68 ± 3.64 eggs/female) were recorded during summer, while the lowest significant rates (194.12 ± 3.59 eggs/female) were observed in winter.

## 5. Conclusions

Seasonal temperature variations are a major factor affecting the developmental duration and growth rate of the forensically important *W. nuba*. Higher temperatures tend to accelerate these processes, while lower temperatures slow them down. An average temperature of 27.9 °C was optimal for achieving the highest level of fecundity. At this temperature, the highest larval and pupal survival rates were observed.

Additionally, the mean lengths and weights of both feeding larvae and wandering prepupae and pupae peaked at an average temperature of 27.9 °C. Obtaining developmental data at various temperatures can provide fundamental information for estimating the age of fly larvae on corpses and accurately estimating the mPMI. Furthermore, even small fluctuations in temperature can impact growth and subsequently affect the estimation of time of death. Therefore, to more precisely determine the time of death, it is essential to consider the historical temperature records of the area where the body was discovered. Future studies could investigate other environmental factors that potentially affect the developmental rates of *W. nuba*.

## Figures and Tables

**Table 1:** The effect of seasonal temperature variations on the duration of immature stages of *Wohlfahrtia nuba*.

Temp. °C (Season) Mean (Range)	Larval Duration (Days) Mean ± SE	Pupal Duration (Days) Mean ± SE (Range)		Total (Mean ± SE)	
		Female	Male	Female	Male
27.9 °C (Spring) (17.11–29.47) 26.97% RH	7.57 ± 0.14 ^a^ (6–10)	12.48 ± 0.23 ^a^ (9–15)	12.59 ± 0.24 ^a^ (9–15)	18.77 ± 0.28 ^a^	18.92 ± 0.29 ^a^
38.3 °C (Summer) (30.66–46.83) 17.23% RH	3.81 ± 0.04 ^b^ (3–4)	8.69 ± 0.12 ^a^ (8–11)	8.88 ± 0.19 ^a^ (7–10)	13.59 ± 0.16 ^b^	14.00 ± 0.35 ^b^
35.8 °C (Autumn) (27.35–43.5) 21.36% RH	4.26 ± 0.06 ^c^ (3–5)	9.43 ± 0.15 ^a^ (8–12)	9.65 ± 0.24 ^a^ (7–11)	12.33 ± 0.13 ^b^	12.68 ± 0.25 ^b^
18.5 °C (Winter) (13.65–22.83) 41.39% RH	10.28 ± 0.07 ^d^ (8–12)	62.57 ± 7.04 ^b^ (39–121)	64.83 ± 5.80 ^b^ (38–119)	72.17 ± 7.09 ^c^	73.50 ± 9.61 ^c^
*p*	0.000	0.000	0.000	0.000	0.000

Means with the same letter in each column are not statistically different at a significance level of *p* < 0.05 based on the LSD test performed after analysis of variance (ANOVA).

**Table 2:** Effect of temperature variations on the growth rate of *Wohlfahrtia nuba* larvae.

Temp. °C (Season) Mean (Range)	Larval Growth Rate (mg/day) (Mean ± SE)
27.9 °C (Spring) (17.11–29.47) 26.97% RH	22.97 ± 0.19 ^a^
38.3 °C (Summer) (30.66–46.83) 17.23% RH	38.61 ± 0.46 ^b^
35.8 °C (Autumn) (27.35–43.5) 21.36% RH	37.11 ± 0.83 ^b^
18.5 °C (Winter) (13.65–22.83) 41.39% RH	13.46 ± 0.52 ^c^
*p*	0.000

Means with the same letter are not statistically different at a significance level of *p* < 0.05 based on the LSD test performed after the analysis of variance (ANOVA).

**Table 3:** Effects of seasonal temperature variations on larval and pupal survival and mortality rates of *Wohlfahrtia nuba*.

Temp. °C (Season) Mean (Range)	Larval Survival (%) Percentage of L1 Reached Pupae (Mean ± SE)	Pupal Survival (%) Percentage of Pupae Reached Adult (Mean ± SE)	Larval Mortality (%) (Mean ± SE)	Pupal Mortality (%) (Mean ± SE)	Mortality Number	
					Larvae	Pupae
27.9 °C (Spring) (17.11–29.47) 26.97% RH	95.75 ± 0.82 ^a^	97.18 ± 0.44 ^a^	4.25 ± 0.82 ^a^	3.18 ± 0.29 ^a^	4 (100)	3 (96)
38.3 °C (Summer) (30.66–46.83) 17.23% RH	58.57 ± 8.96 ^b^	81.64 ± 0.72 ^b^	41.43 ± 8.96 ^b^	10.92 ± 0.33 ^b^	41(100)	11 (59)
35.8 °C (Autumn) (27.35–43.5) 21.36% RH	74.57 ± 5.77 ^c^	90.64 ± 0.77 ^c^	25.43 ± 5.77 ^c^	6.92 ± 0.47 ^c^	25 (100)	7 (75)
35.8 °C (Autumn) (27.35–43.5) 21.36% RH	74.57 ± 5.77 ^c^	90.64 ± 0.77 ^c^	25.43 ± 5.77 ^c^	6.92 ± 0.47 ^c^	25 (100)	7 (75)
18.5 °C (Winter) (13.65–22.83) 41.39% RH	91.73 ± 2.02 ^a^	45.73 ± 0.99 ^d^	9.36 ± 8.59 ^d^	45.67 ± 0.49 ^d^	9 (100)	46 (92)
*p*	0.000	0.000	0.000	0.000		

Means with the same letter in each column are not statistically different at a significance level of *p* < 0.05 based on the LSD test performed after analysis of variance (ANOVA).

**Table 4:** The effects of temperature variations on the weight and length of larval instars, prepupae, and pupae of *Wohlfahrtia nuba*.

Temp. °C (Season) Mean (Range)	Developmental Stage Weight (mg) (Mean ± SE)					Developmental Stage Length (mm) (Mean ± SE)				
	Feeding Larvae			**PrePupae**	Pupae	Feeding Larvae			**Pre Pupae**	Pupae
	First Instar	Second Instar	Third Instar			First Instar	Second Instar	Third Instar		
27.9 °C (Spring) (17.11–29.47) 26.97% RH	17.49 ± 0.36 ^a^	111.25 ± 3.92 ^a^	191.82 ± 1.54 ^a^	124.95± 2.56 ^a^	109.71 ± 2.57 ^a^	6.07 ± 0.31 ^a^	18.82 ± 0.49 ^a^	26.11 ± 0.33 ^a^	22.44 ± 0.32 ^a^	13.91 ± 0.29 ^a^
38.3 °C (Summer) (30.66–46.83) 17.23% RH	15.24 ± 0.37 ^b^	90.50 ± 2.11 ^b^	160.53± 2.47 ^b^	105.0 ± 3.45 ^b^	91.57 ± 2.12 ^b^	4.93 ± 0.36 ^b^	17.39 ± 0.52 ^b^	24.33± 0.46 ^b^	21.39 ± 0.27 ^b^	12.98 ± 0.19 ^b^
35.8 °C (Autumn) (27.35–43.5) 21.36% RH	16.66 ± 0.28 ^a^	97.75 ± 2.22 ^b^	178.06 ± 4.29 ^c^	117.26± 4.29 ^c^	99.95 ± 9.89 ^c^	5.14 ± 0.33 ^b^	17.71 ± 0.58 ^b^	25.11 ± 0.46 ^a^	21.89 ± 0.29 ^b^	13.25 ± 0.35 ^a^
18.5 °C (Winter) (13.65–22.83) 41.39% RH	12.41 ± 0.41 ^c^	70.47 ± 4.06 ^c^	141.41 ± 4.02 ^d^	104.57± 3.36 ^b^	88.25 ± 2.72 ^d^	4.29 ± 0.39 ^b^	16.18 ± 0.37 ^c^	22.82 ± 0.38 ^c^	18.72 ± 0.39 ^c^	12.57 ± 0.20 ^b^
*p*	0.000	0.000	0.000	0.000	0.000	0.007	0.002	0.000	0.000	0.000

Means with the same letter in each column are not statistically different at a significance level of *p* < 0.05 based on the LSD test performed after analysis of variance (ANOVA).

**Table 5:** The effect of temperature variations on the number and percentage of emergence of females and males, sex ratio, pre-larviposition period, and fecundity of *Wohlfahrtia nuba*.

Temp. °C (Season) Mean (Range)	Number of Adults Emerged (Mean ± SE)		% Emergence		Sex Ratio		Pre-Larviposition Period	No. of Larvae Laid/Female (Fecundity) (Mean ± SE) (Range)
	Female	Male	Female	Male	Female	Male		
27.9 °C (Spring) (17.11–29.47) 26.97% RH	55.67 ± 0.56 ^a^	37.17 ± 0.87 ^a^	60	40	1.50	1	7.83 ± 0.35 ^a^	51.42 ± 1.22 ^a^ (43–55)
38.3 °C (Summer) (30.66–46.83) 17.23% RH	26.83 ± 0.75 ^b^	20.83 ± 0.79 ^b^	56.25	43.75	1.29	1	4.17 ± 0.31 ^b^	42.08 ± 0.65 ^b^ (39–45)
35.8 °C (Autumn) (27.35–43.5) 21.36% RH	39.67 ± 0.80 ^c^	31.17 ± 0.84 ^c^	57.35	45.59	1.26	1	5.76 ± 0.41 ^b^	45.83 ± 0.52 ^c^ (43–48)
18.5 °C (Winter) (13.65–22.83) 41.39% RH	23.83 ± 0.54 ^d^	14.67 ± 1.12 ^d^	61.54	38.462	1.60	1	11.25 ± 0.33 ^c^	39.58 ± 0.34 ^d^ (38–41)
*P*	0.00	0.00					0.00	0.00

Means with the same letter in each column are not statistically different at a significance level of *p* < 0.05 based on the LSD test performed after the analysis of variance (ANOVA).

## Data Availability

The original contributions presented in this study are included in the article. Further inquiries can be directed to the corresponding author.

## Abbreviations

The following abbreviations are used in this manuscript:

PMI	Post-mortem interval
mPMI	Minimum post-mortem interval
LGR	Larval growth rate
P	Pupa
